# Perinatal Neuroprotective Strategies and Intraventricular Hemorrhage in Preterm Infants ≤32 Weeks’ Gestational Age: A Prospective Study With a Historical Control Cohort

**DOI:** 10.7759/cureus.109728

**Published:** 2026-05-27

**Authors:** Anu Sharma, Radhika Sujatha, Krishna Nesseril, Sobha Kumar

**Affiliations:** 1 Neonatology Division, Department of Pediatrics, Institute of Medical Sciences, Banaras Hindu University, Varanasi, IND; 2 Department of Neonatology, Government Medical College, Thiruvananthapuram, Keralam, IND; 3 Department of Neonatology, Government Medical College, Ernakulam, Keralam, IND

**Keywords:** cystic periventricular leukomalacia, intraventricular hemorrhage, ivh care bundle, neuroprotective strategies, preterm infants

## Abstract

Background

Intraventricular hemorrhage (IVH) is a major complication of prematurity and a significant contributor to increased morbidity and mortality. There is no single intervention to eliminate IVH; however, implementing perinatal neuroprotective strategies (NPS) may help minimize adverse outcomes. Indian data on perinatal NPS and their association with IVH remains limited.

Purpose

The aim of this study was to compare IVH incidence before and after implementation of perinatal NPS (antenatal neuroprotection and postnatal IVH care bundle) in preterm infants ≤32 weeks’ gestational age (GA). Secondary objectives were to identify risk factors associated with IVH and to determine the incidence of cystic periventricular leukomalacia (cPVL).

Methods

Baseline incidence of IVH was reviewed retrospectively from case records of the year 2019 among all preterm infants ≤32 weeks’ GA. During the study period (July 2020 to December 2021), preterm infants ≤32 weeks’ GA who received NPS during the antenatal (antenatal steroids and magnesium sulfate) and postnatal (IVH care bundle) periods were included. Assessment for IVH and cPVL was performed using neurosonography during the hospital stay and at 38-40 weeks’ GA, respectively.

Results

A total of 421 preterm infants were included. IVH incidence was similar in the 2019 baseline cohort and during July 2020 to December 2021 (71/414 [17.15%] vs 63/421 [14.96%]; OR 1.18, CI 0.81-1.70, p = 0.39). Mean GA and mean birth weight (BW) were 30 week + 1 day (SD ±1.7) and 1,217.19 g (SD ±309.4), respectively. The overall incidence of IVH was 63/421 (14.96%), and the incidence of severe IVH (grade ≥ 3) was 9/421 (2.14%). On logistic regression analysis, GA<28 weeks (adjusted odds ratio [aOR] 2.44), BW < 1,000 g (aOR 3.61), and acidosis (aOR 2.65) were associated with IVH. The incidence of cPVL was 21/348 (6.03%).

Conclusion

Perinatal NPS did not influence IVH incidence; however, the absence of a concurrent parallel comparison group limits inference. The incidence of severe IVH (grade ≥ 3) remained low. Extreme prematurity, extremely low BW, and metabolic acidosis were key factors associated with IVH.

## Introduction

Intraventricular hemorrhage (IVH) is an important cause of increased mortality and morbidity in preterms. The majority of IVH occurs in the first three to four days of life. Incidence is inversely related to gestational age (GA). A recent meta-analysis reported IVH incidences of 17.4% in very preterm infants and 34.3% in extremely preterm infants, with severe IVH in around one-third to one-half of cases [1,2]. Increasing severity is associated with neurological sequelae such as post-hemorrhagic hydrocephalus (PHH), periventricular leukomalacia (PVL), cerebral palsy, global developmental delay, and mortality, which, in turn, lead to stress on families as well as increased financial burden [3,4].

Fragile germinal matrix, immaturity of cerebral vasculature, fluctuating cerebral blood flow (CBF), platelet and coagulation disorders, and placental hemodynamics are important contributors to IVH [5]. Rapid intravenous boluses, fluctuating carbon dioxide values, hemodynamic instability, and excessive handling of neonates cause a rapid increase in CBF, thereby predisposing immature cerebral vasculature to rupture and resulting in IVH. Although there is no single intervention to eliminate IVH, implementing perinatal neuroprotective strategies (NPS) may help minimize adverse outcomes. Among NPS, antenatal corticosteroids (ANS) are beneficial in reducing IVH [6]. Delayed cord clamping (DCC) results in the stabilization of blood pressure at birth and avoids abrupt pressure change, thereby reducing IVH risk [7]. Midline head positioning during the immediate postnatal period maintains a constant CBF and helps reduce impedance to venous flow. In addition, minimal handling and avoidance of stressful situations may be beneficial in decreasing IVH incidence [8,9]. Magnesium sulfate (MgSO4) is known to decrease the risk of preterm white‑matter injury via several neuroprotective mechanisms, including antioxidant effects and membrane stabilization, and is used to improve neurodevelopment outcome independent of IVH reduction [10].

Published literature on the effectiveness of NPS and IVH care bundle (ICB) from the developed world outnumber data from low- and middle-income countries. Furthermore, there are no data from India on perinatal NPS, their implementation, and their association with IVH. Therefore, this study aimed to compare IVH incidence before and after the implementation of perinatal NPS in preterm infants ≤32 weeks’ GA. Secondary objectives were to identify risk factors associated with IVH and to determine the incidence of cystic periventricular leukomalacia (cPVL).

## Materials and methods

This prospective cohort study was conducted in the Department of Neonatology, Government Medical College (GMC), Thiruvananthapuram, Keralam, India, from July 2020 to December 2021. Retrospective baseline incidence of IVH was reviewed from case records of the year 2019 among all preterm infants ≤ 32 weeks’ GA. ICB was implemented in the unit two months prior to starting this study as a routine care protocol for the first five days of life to prevent preterm IVH. During the study period, preterm infants ≤ 32 weeks’ GA whose mothers received a complete course of ANS and MgSO_4_ (antenatal neuroprotection) or, if not complete, at least 24 hours’ duration of ANS and 6 hours of MgSO_4_, were included. ANS was given as dexamethasone 6 mg intramuscular (IM) 12 hours apart for 48 hours or betamethasone 12 mg IM 24 hours apart for 48 hours, and repeat course was given if delivery had not occurred within seven days after the initial dose. MgSO_4_ neuroprotection was given intravenously as 4 g/kg loading dose over 30 minutes, followed by 1 g/kg/h for 24 hours or till birth, whichever was sooner. DCC (60-second duration) was performed in babies who had a stable cardiorespiratory status at birth. Postnatal care included application of established ICB in the first five days (Table 1).

**Table 1:** Components of ICB ICB, IVH care bundle; IV, intravenous; PCO₂, partial pressure of carbon dioxide

ICB (for the first 120 hours of life)
1. Maintain a neutral head position and elevate the head to 15°-30°.
2. Avoid raising feet above the head during nursing care (diaper change).
3. No daily weight monitoring until day 5.
4. Measured suction (endotracheal, deep nasal/oral) and only when clinically indicated.
5. Avoid rapid IV boluses.
6. Avoid hypercarbia and rapid fluctuation in PCO_2_.

Preterm infants with congenital malformations, intrauterine infections, intrauterine IVH, and GA < 25 weeks were excluded; those who died in the first 24 hours of life were also excluded. During the study period, monthly meetings were conducted for the NICU staff and residents for sustaining ICB practices. No process or outcome indicators were assessed to evaluate compliance of ICB.

The study was approved by the Institutional Review and Ethical Board. Data collected from prospectively enrolled patients included baseline maternal and neonatal demographics. In addition to the aforementioned demographics, we also collected data on neonatal risk factors for IVH, such as the need for neonatal resuscitation, invasive mechanical ventilation, inotrope use and hypercarbia (PCO_2_ > 60 mmHg) on blood gas within five days, and the use of surfactant therapy. Relationship between severe metabolic acidosis (BE > -12 mmol/L), thrombocytopenia (platelet count <1 lakh/uL) within the first five days, hemodynamically significant patent ductus arteriosus (hsPDA), blood culture positive sepsis, and necrotizing enterocolitis (NEC) stage ≥ Ⅱ during the hospital stay was also explored.

Neurosonography was performed by a neonatologist trained in point-of-care ultrasound (POCUS) at one, three, and seven days [11]. Scans were performed more often based on clinical conditions or any catastrophic event, such as seizures, or frequent apnea. Papile’s classification system was used for grading IVH [12]. Grades I and II were categorized as mild IVH, and grades III and periventricular hemorrhagic infarction (PVHI) were categorized as severe IVH. Scanning was also performed in all preterm infants at 38-40 weeks’ GA to determine the incidence of cPVL. PVL was classified based on the De Vries grading system [13].

Baseline incidence of IVH at our institute was 17%, which was similar in another Indian study [14]. To reduce the baseline incidence to 10% based on available literature with a power of 0.80, alpha of 0.05, and attrition rate of 10%, 421 preterm infants were included. Descriptive statistics were applied, where categorical variables were reported as frequencies and percentages and continuous variables were reported as means with standard deviations. The chi-square test and Fisher’s exact test were used for comparing IVH incidence pre- and post-implementation period (2019 vs 2020-2021) and for assessing maternal and neonatal risk factors associated with IVH. A multivariate logistic regression model was applied for variables reported to be statistically significant on univariate analysis and to adjust for confounders. Data entry was done using Microsoft Excel (Microsoft Corp., Redmond, WA), and analysis was conducted using SPSS 26 (IBM Corp., Armonk, NY). The level of significance was taken at p < 0.05.

## Results

**Figure 1:**
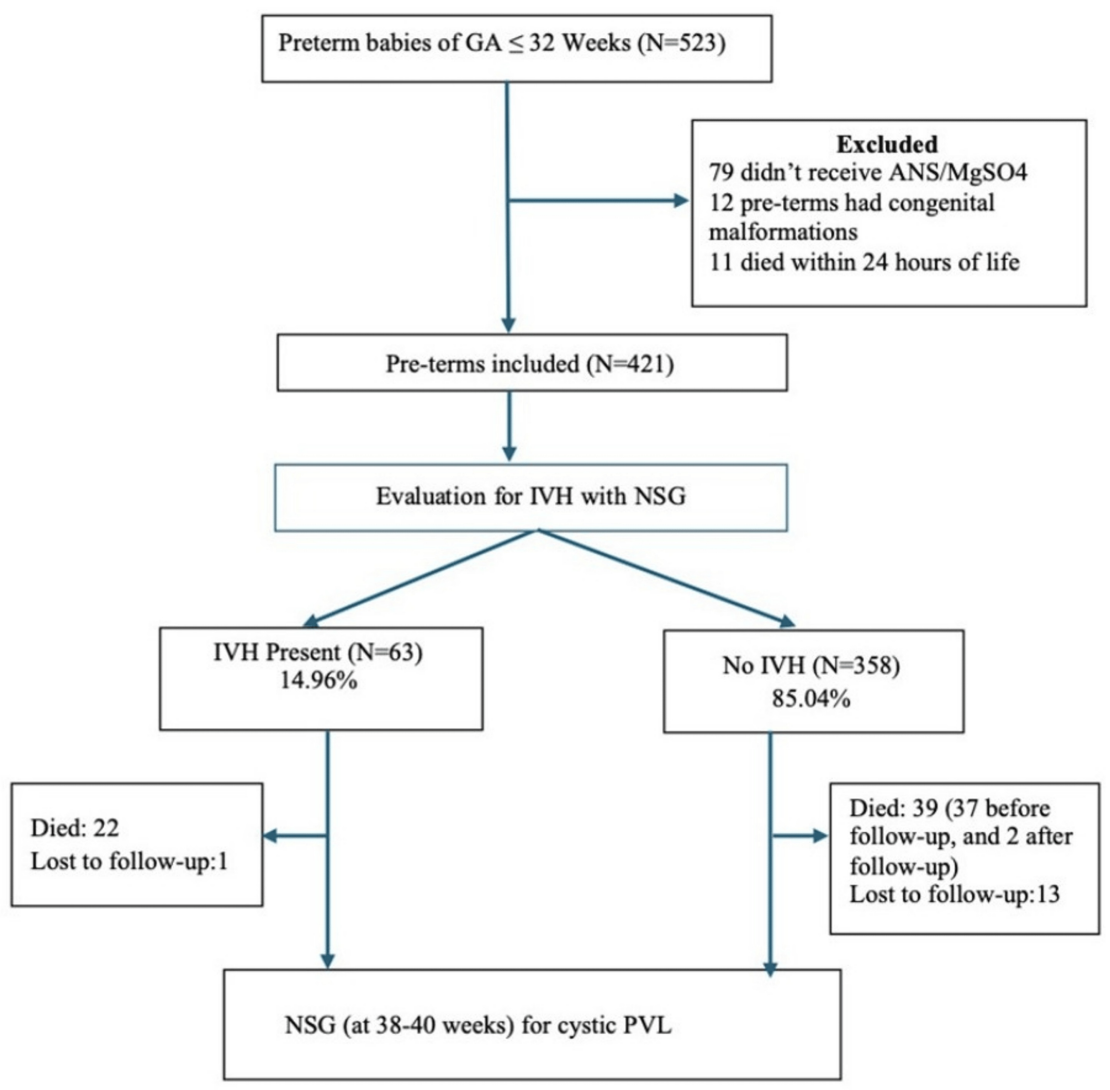
Process flow chart of the study population ANS, antenatal corticosteroids; IVH, intraventricular hemorrhage; MgSO_4_, magnesium sulphate; NSG, neurosonography; PVL, periventricular leukomalacia

**Figure 2:**
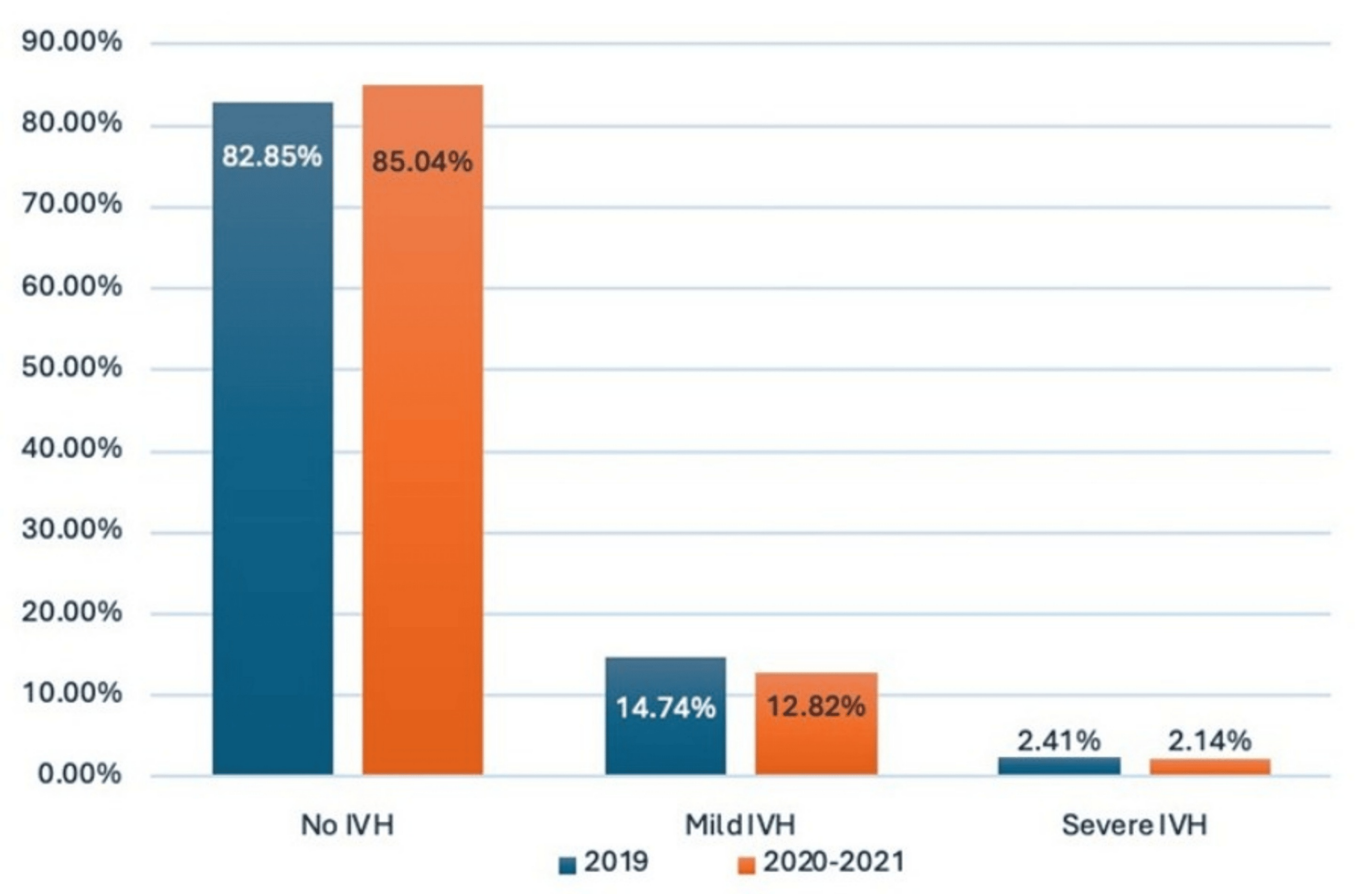
Trends in the IVH incidence among preterm infants before and after perinatal neuroprotective strategies IVH, intraventricular hemorrhage; mild IVH, grade 1 and grade 2; severe IVH, grade 3 and periventricular hemorrhagic infarction

Participant flow is summarized in Figure 1. The baseline (pre‑implementation) phase comprised a retrospective cohort of preterm infants ≤32 weeks’ GA admitted in 2019. This was followed by the post‑ICB implementation phase, which comprised a prospective cohort enrolled during July 2020 to December 2021. The incidence of any IVH was 71/414 (17.15%) in 2019 and 63/421 (14.96%) in 2020-2021 (OR: 1.18, 95% CI: 0.81-1.70, p = 0.39), whereas the incidence of severe IVH (grade ≥ 3) was 10/414 (2.41%) and 9/421 (2.14%), respectively (OR: 1.13, 95% CI: 0.46-2.82, p = 0.82) (Figure 2).

**Figure 3:**
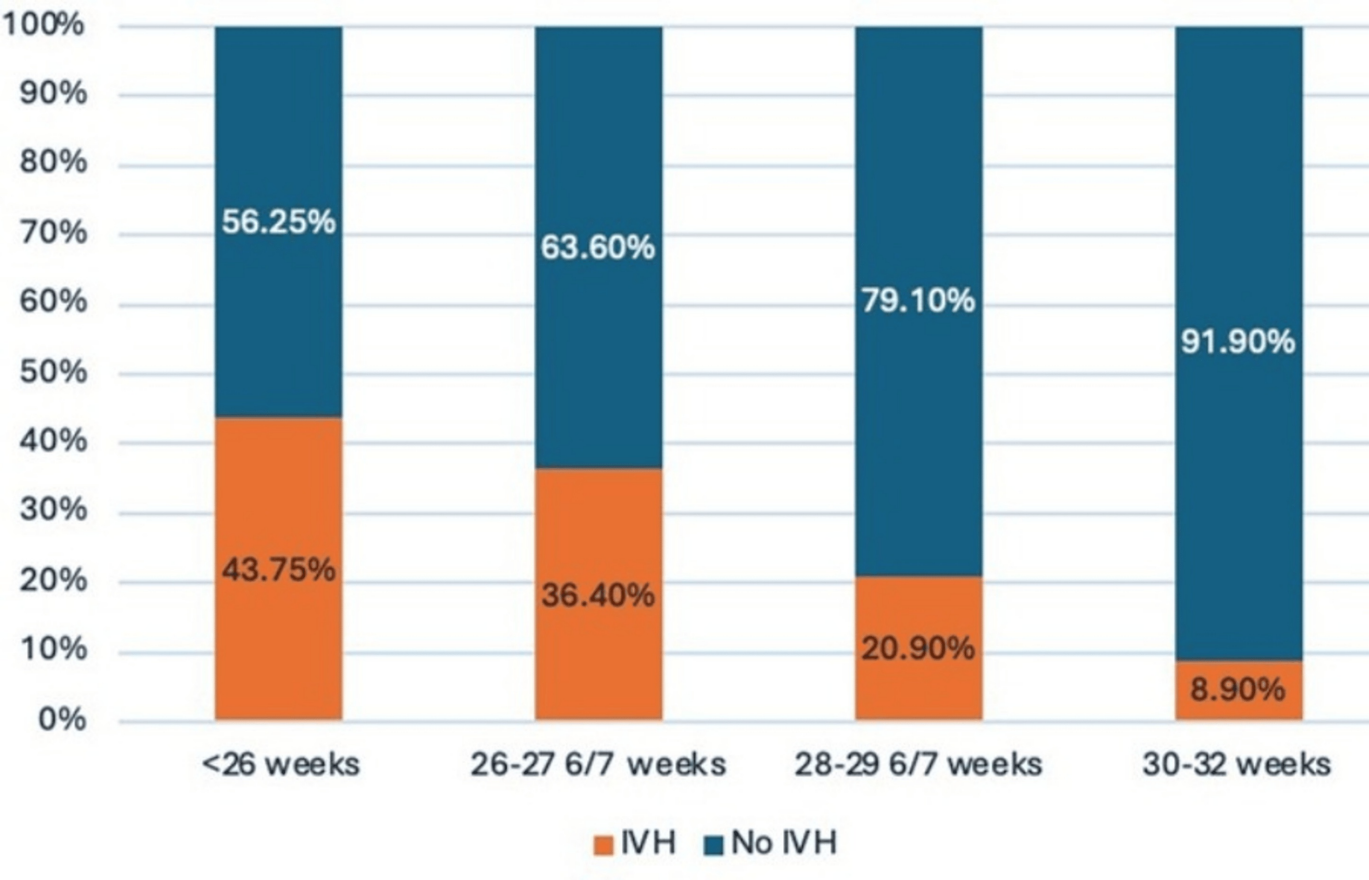
Percentage distribution of babies with and without IVH according to gestational age IVH, intraventricular hemorrhage

A total of 421 preterm infants were included in the prospective phase. Mean GA and mean birth weight (BW) were 30 weeks ± 1 day (SD ±1.7) and 1,217.19 g (SD ±309.4), respectively. One-quarter of the patients had an extremely low BW (Table 2). IVH incidence was highest among infants < 26 weeks’ GA, 7/16 (43.75%). The observed higher incidence may reflect a small sample size in the subgroup <26 weeks’ GA (Figure 3).

**Table 2:** Baseline characteristics of the study population APH, antepartum hemorrhage; GA, gestational age; hsPDA, hemodynamically significant patent ductus arteriosus; IMV, invasive mechanical ventilation; LSCS, lower segment cesarean section; NEC, necrotizing enterocolitis; NNR, neonatal resuscitation; PPROM, preterm premature rupture of membranes; SGA, small for gestational age

Variable	Mean ± SD or N (%)
Mean GA	30.17 (SD±1.7)
Mean birth weight	1217.19 (SD±309.4)
GA < 28 weeks	49 (11.63%)
Birth weight < 1,000 g	109 (25.89%)
Male	242 (57.48%)
SGA	129 (30.64%)
LSCS	284 (67.45%)
Antenatal factors	
Twin/triplet pregnancy	129 (30.64%)
Maternal infections (urosepsis + chorioamnionitis + PPROM)	148 (35.15%)
Abnormal Doppler	49 (11.63%)
APH	11 (2.61%)
Hypertension/eclampsia	87 (20.66%)
Perinatal factors	
Need for NNR	121 (28.74%)
Need for surfactant	184 (43.70%)
IMV	149 (35.39%)
Metabolic acidosis	102 (24.22%)
hsPDA	67 (15.91%)
Inotropes requirements	89 (21.14%)
Blood culture positive sepsis	109 (25.89%)
NEC stage ≥ II	79 (18.76%)
Platelet <1 lakh in the first seven days of life	75 (17.81%)

On univariate analysis, need for NNR in the delivery room, lack of DCC, surfactant use, inotrope use during the first five days, metabolic acidosis, thrombocytopenia (platelet count < 1 lakh/uL) within the first five days, hsPDA, and NEC stage ≥ II (p = 0.000) were significantly associated with IVH. Blood culture-positive sepsis (p = 0.001) and need for IMV (p < 0.001) were also associated with higher IVH incidence (Table 3). Variables such as hsPDA, NEC stage ≥ II, and culture-positive sepsis may occur after IVH onset and reflect overall disease severity rather than the actual temporal relationship. On logistic regression analysis, we found GA < 28 weeks (adjusted odds ratio [aOR] : 2.44, 95% CI: 1.17-5.07), BW < 1,000 g (aOR 3.61, 95% CI: 1.95-6.66), and metabolic acidosis (aOR: 2.650, CI: 1.318-5.326, p = 0.006) to be associated risk factors for IVH (Table 4).

**Table 3:** Maternal and neonatal risk factors for IVH *Abnormal Doppler, no DCC, need for NNR in delivery room, GA < 28 weeks, BW < 1,000 g, surfactant administration, IMV, inotrope use, metabolic acidosis, blood culture-positive sepsis, platelet count < 1 lakh/uL, and NEC stage ≥ II all had a significant association with IVH on univariate analysis. Mortality was higher in the IVH group. DCC, delayed cord clamping; GA, gestational age; hsPDA, hemodynamically significant patent ductus arteriosus; IMV, invasive mechanical ventilation; IVH, intraventricular hemorrhage; LSCS, lower segment cesarean section; NEC, necrotizing enterocolitis; NNR, neonatal resuscitation; PCO_2_, partial pressure of carbon dioxide

Maternal Factors	IVH Present (63)	No IVH (358)	Total (421)	RR (95% CI)	p-Value
LSCS	37 (58.73%)	247 (69.00%)	284	1.45 (0.92-2.3)	0.107
Maternal infections	16 (25.40%)	132 (36.87%)	148	0.63 (0.37-1.0)	0.079
Hypertension/eclampsia	12 (19.04%)	75 (21.00%)	87	0.9 (0.5-1.61)	0.731
Abnormal Doppler	12 (19.04%)	37 (10.33%)	49	1.78 (1.02-3.1)	0.047*
Neonatal factors					
No DCC	42 (66.66%)	135 (37.70%)	177	2.75 (1.7-4.5)	0.000*
Need for NNR	30 (47.61%)	91 (25.41%)	121	2.25 (1.4- 3.5)	0.000*
GA < 28 weeks	19 (30.15%)	30 (8.38%)	49	3.27 (2.1-5.1)	0.000*
Birth weight < 1,000 g	35 (55.55%)	74 (20.67%)	109	3.57 (2.28-5.59)	0.000*
Male	41 (65.07%)	201 (56.14%)	242	1.37 (0.85-2.2)	0.186
Need for surfactant	45 (71.42%)	139 (38.82%)	184	3.21 (1.93-5.37)	0.000*
IMV	48 (76.19%)	101 (28.21%)	149	5.84 (3.38-10.1)	0.000*
Use of inotropes	35 (55.55%)	54 (15.08%)	89	4.67 (3.0-7.24)	0.000*
Hypercarbia (pCO_2_ > 60 mmHg)	2 (3.17%)	16 (4.46%)	18	0.27 (0.19-2.77)	0.896
Metabolic acidosis within 5 days	36 (57.14%)	66 (18.43%)	102	4.16 (2.66-.53)	0.000*
Blood culture-positive sepsis	27 (42.85%)	81 (22.62%)	109	2.17 (1.38-3.4)	0.001*
Platelet < 1 lakh/uL within 5 days	25 (39.68%)	50 (13.96%)	75	3.03 (1.95-4.71)	0.000*
NEC stage ≥ II	27 (42.85%)	52 (14.52%)	79	3.21 (2.08-4.97)	0.000*
Mortality during the hospital stay	22 (34.92%)	39 (10.90%)	61	3.16 (2.04-4.92)	<0.001*

**Table 4:** Logistic regression analysis of risk factors associated with IVH *Gestational age < 28 weeks, birth weight < 1,000 g, and metabolic acidosis were significant risk factors associated with IVH. aOR, adjusted odds ratio; IVH, intraventricular hemorrhage

Variable	aOR	95% CI	p-Value
Gestational age < 28 weeks	2.44	1.17-5.07	0.017*
Birth weight < 1,000 g	3.61	1.95-6.66	0.001*
Presence of metabolic acidosis	2.650	1.318-5.326	0.006*

Of the 348 preterm infants who were followed up at 38-40 weeks’ GA, 21/348 (6.03%) had cPVL. This may not be reflective of actual incidence, given that 61/421 (14.50%) died before follow-up (Figure 1). One-third (33.33%) of preterm infants who were found to have cPVL at 38-40 weeks’ GA had also suffered from IVH during NICU stay (RR: 3.85, 95% CI: 1.65-8.96; p = 0.001) (Table 5).

**Table 5:** Incidence of cPVL at 38–40 weeks’ gestational age and its association with IVH *One-third of patients with cPVL had experienced IVH during the early neonatal period. cPVL, cystic periventricular leukomalacia; IVH, intraventricular hemorrhage

Variable	cPVL	No cPVL	RR (95% CI)	p-Value
Overall incidence	21/348=6.03%	327/348=94.00%	-	-
IVH				
Yes	7 (33.33%)	33 (10.09%)	3.85 (1.65-8.96)	0.001*
No	14 (66.66%)	294 (89.90%)		

## Discussion

Following the implementation of perinatal NPS, the incidence of IVH was not significantly different from the pre-implementation period. However, this finding may not necessarily be interpreted as the absence of effect, given the lack of a parallel concurrent control group. Maintaining a low incidence of severe IVH is clinically important because high-grade IVH is strongly associated with mortality and long-term neurodevelopmental impairment. The low baseline rate of severe IVH and the small number of severe events may also have limited our ability to detect modest changes in severe IVH following NPS implementation. There is no published evidence from India on IVH incidence post-NPS implementation.

Similar to ours, no reduction in IVH was observed by Gross et al. in preterm infants with GA < 30 weeks and BW < 1,250 g after application of the neonatal care bundle [15]. The author concluded that the beneficial effects of interventions in one NICU do not necessarily lead to similar results, as local NICU practices, patient profiles, bundle components, and adherence may vary and require a more individualized approach, and emphasized the need for a larger randomized multicenter trial to evaluate the effectiveness of a neuro care bundle on IVH.

Schmid et al. observed a reduction in the incidence of PIVH from 22.1% to 10.5% and that of severe IVH from 9.1% to 3.7% among very low birth weight (VLBW) infants after utilizing preventive measures during the perinatal period [16]. Furthermore, any grade IVH post-intervention was 49%, and severe grades were 6%, as reported by Wallau et al. [17]. As 28.2% of their study cohort had GA< 28 weeks and their sample size was small compared to ours, this may explain the higher IVH incidence. Despite the higher IVH incidence, the authors reported a statistically significant reduction and suggested that care practices that minimize fluctuations in CBF among VLBW preterm infants in the immediate newborn period might have a beneficial effect on neuromorbidity [17]. Another study observed a significant decrease in “new or progressive IVH” within the first 72 hours after birth (aOR: 0.34; 95% CI: 0.20-0.56; p < 0.001) as well as in their primary composite outcome, “new or progressive IVH, mortality, or cPVL” (aOR 0.42; 95% CI 0.2-0.65; p < 0.001), and reported a positive impact of a nursing care bundle on IVH incidence [9]. We also applied ICB during the initial transition period when preterms are more prone to hemodynamic compromise and CBF fluctuation. In addition to ICB, we also included antenatal neuroprotection in our preterm infants. Discrepancies in results may be attributed to study design and/or differences in care settings. ANS, MgSO4 neuroprotection, DCC, and measures to stabilize CBF have also been included in the ICB development study by Mohammedi and Miall [18]. The presence of a round-the-clock neonatologist and changes in unit policy significantly decreased IVH from 9.4% in 2016 to 5% in 2018 (p = 0.044) in one study [19].

Cesarean section is observed to be a protective factor for the occurrence of any grades of IVH (aOR 0.166, 95% CI 0.053-0.515) [17]. A study from Saudi Arabia reported that cesarean section was protective for severe IVH in their cohort [19], while another reported an association of vaginal delivery with severe IVH (RR=0.42, 95% CI: 0.28-0.63) [20]. This association was not observed in our study. Higher IVH incidence is also observed in amniotic infection syndrome [15,17]. Similar to the findings of Vogtmann et al. and Singh et al., there was no gender difference in IVH incidence in our preterm infants [21,22]. However, other studies have described a higher incidence of severe IVH in male infants [23,24]. On univariate analysis, most of our findings of neonatal factors were similar to those reported in the literature [25-27].

On regression analysis of neonatal characteristics, GA < 28 weeks, BW < 1,000 g, and metabolic acidosis were associated with the occurrence of IVH. The possibility of multicollinearity should be considered as GA and BW are highly correlated variables. Published literature reports higher IVH incidence in patients who require early intubation within 72 hours of life (aOR 12.6), have thrombocytopenia (platelet count <1.5 lakh/uL in the first week) (aOR 3.134), and have hypotension needing inotropes (aOR 4.664) [15,17].

We found a 6.03% incidence of cPVL at 38-40 weeks’ corrected age, which is higher than that reported in the literature (1.0% in de Bijl-Marcus et al.’s study, 0.5% in Schmid et al.’s study, 3.7% in Wang et al.’s study) [9,16,28]. Differences in neuroimaging modality, its timing, and patient profile may account for variation in cPVL incidence across studies. Neurologic injury, as an outcome of grade III/IV IVH and PVL, was observed in 15% of patients in a recent report by Lee et al. following the implementation of NPS [29]. In line with ours, this was the only study where authors assessed the impact of NPS applied during both the antenatal and postnatal periods on PVL.

Strengths and limitations

Strengths of this study include being the first prospective study from a tertiary-level public sector setting in India on perinatal NPS and their association with IVH incidence in preterm infants ≤ 32 weeks’ GA, conducted on a fairly large sample size. This study underscores the importance of implementing standard perinatal NPS in resource-constrained settings, even when clear benefits are not observed, as outcomes may vary due to multifactorial etiologies, local NICU conditions, and patient profile.

Our study had several limitations. As this was a pre-post observational design without a concurrent parallel control group, it is difficult to attribute any change (or lack of change) in IVH incidence solely to NPS. Baseline data were drawn retrospectively from clinical records, which may under-ascertain IVH compared with prospectively observed incidence, introducing potential confounding. In addition, the pre-post comparisons were unadjusted and may be influenced by secular trends and other time-varying factors such as changes in NICU practices, pandemic-related healthcare alterations, and patient characteristics. The study was also likely underpowered to detect small differences in severe IVH. Exclusion of patients who died within the first 24 hours may result in selection bias. Adherence to ICB was not documented. Ideally, an MRI of the brain is required to study the extent of preterm brain injury and non-cystic PVL, which we could not perform. As our intervention was a bundle, the impact of individual components could not be assessed.

## Conclusions

IVH incidence during July 2020 to December 2021 was similar to the 2019 retrospective baseline incidence; however, the lack of a concurrent comparison group limits causal inference. Notably, severe IVH remained low, which is clinically important given the high mortality and neuromorbidity in infants with severe IVH. Perinatal NPS still remain a practical approach to offer neuroprotective care during the early, vulnerable neonatal period, and future efforts should focus on systematic documentation of bundle adherence and adaptations tailored to local settings to optimize patient outcomes.
